# Violent and Nonviolent Youth Offenders: Preliminary Evidence on Group Subtypes

**DOI:** 10.1177/1541204015615193

**Published:** 2015-11-20

**Authors:** Violet Lai, Gerald Zeng, Chi Meng Chu

**Affiliations:** 1 Research for Evidence-based Practice, Social Service Institute, Singapore; 2 Centre for Research on Rehabilitation and Protection, Ministry of Social and Family Development, Singapore

**Keywords:** violence, youth, type, risk, needs, recidivism

## Abstract

Youth violence is a costly social problem. This study compared the risk and needs of nonviolent youth offenders, with those who had committed violent offenses only (violent only) and those who had committed violent and nonviolent offenses (violent plus) to determine whether violent youth were a different “type” from nonviolent youth. The case files of 3,744 youth offenders (3,327 males and 417 females, between 12 and 18 years old) were retrospectively coded, before official recidivism records were obtained. Multivariate analysis of variance (MANOVA), χ^2^, and Cox regressions were conducted. Violent-plus youth were younger; higher in their total risk and all criminogenic needs; more likely to have several noncriminogenic needs; and at higher risk of any reoffending, violent reoffending, and nonviolent reoffending than nonviolent youth. Violent-only youth had the same total risk and risk of general and violent recidivism as nonviolent offenders but presented different criminogenic and noncriminogenic needs and risk of nonviolent recidivism. Compared to violent-only youth, violent-plus youth were younger, had higher total risk and criminogenic needs on five domains, were more likely to have several noncriminogenic needs, and were at higher risk of all types of reoffending (except sexual reoffending), suggesting subtypes of violent youth offenders. The implication is that nonviolent and violent youth offenders require different dosage and types of intervention.

## Introduction

Youth violence is one of the most prominent social problems (Davies & Pearson, 1999; Mercy, Butchart, Farrington, & Cerda, 2002). Studies in United States and United Kingdom estimated that the tangible and intangible costs of violent crimes, which include those committed by youth, amounted to billions per year (Institute for Economics & Peace, 2013; Shapiro & Hassett, 2012). Notwithstanding the volatility of youth violence trends (Butts & Snyder, 2006; Cook & Laub, 2002), the substantial costs of each violent act (McCollister, French, & Fang, 2010) confirm that violent offending by youth requires attention. By virtue of their young age, persistent violent offending by youth can result in longer term costs and potentially be more damaging when compared to adult violence. For the above-mentioned reasons, a better understanding of youth violence is critical for advancing prevention and intervention efforts.

### Typological Distinction Between Violent and Nonviolent Youth Offenders

Offender typology involves classifying offenders into theoretically meaningful or practical categories, on the basis that not all offenders are the same (Clements, 1996; Moffitt, 1993). Although the development of reliable and valid offender typologies has its challenges (Byrne & Roberts, 2007), offender categories are still useful in understanding offending behavior and in predicting reoffending (Schmidt, Campbell, & Houlding, 2011). It has also been successfully applied in offender treatment and management, for example, a meta-analysis showed that matching the intensity of treatment programs to offenders’ risk categories was effective in reducing recidivism (Andrews & Dowden, 2006).

There is a substantial volume of past research on typologies of delinquents (see brief review by Loeber, Farrington, & Waschbusch, 1998). In particular, Loeber and colleagues examined a group of young offenders termed as “serious violent juvenile offenders.” By identifying their unique risk and protective factors, developmental trajectories, and criminal careers, from longitudinal studies using self-reports and official records, their research put forward violent delinquents as a category separate from general delinquency (Loeber & Farrington, 1998).

Recent research has continued to uncover evidence for a violent group of youth offenders, across different operationalization of violent behavior, some of which included combinations with other offense types (e.g., violent substance abuser) and offender characteristics (e.g., violent chronic offender). Using a large, representative sample of 18,614 adolescents, Vaughn, Salas-Wright, DeLisi, and Maynard (2014) identified a severe 5% group who not only showed higher levels of violence but also higher levels of substance abuse, and other delinquency behaviors. Baglivio, Jackowski, and Greenwald (2014) used an actuarial risk assessment instrument to characterize serious violent chronic (SVC) youth offenders and found that their reoffending was predicted by a different set of risk and protective factors compared to the non-SVC youth offenders. Another study found that the risk factors that predicted the severity of reoffending were different for serious violent offenders, violent property offenders, property offenders, and sex offenders (Mulder, Vermunt, Brand, Bullens, & Van Marle, 2012). These studies were conducted in Western countries, with majority of the samples comprising youth of non-Asian descent.

Although there are overlaps across cultures on their definitions of criminal behavior, the development of deviant behaviors can differ due to cultural norms, morals, religion, taboos, and expectations (e.g., Bhugra, Popelyuk, & McMullen, 2010; Lahlah, Van der Knaap, Bogaerts, & Lens, 2013). For example, studies showed that cultural factors are related to the risk and protective factors of violent offending (e.g., Flanagan et al., 2011; Soriano, Rivera, Williams, Daley, & Reznik, 2004). Given the lack of research on violent typology among Asian youth offenders, it is useful to examine the dichotomy of violent and nonviolent offenders along with the risk and needs associated with each group in this population.

The distinction between violent and nonviolent offenders has important practical implications for law enforcers and rehabilitation practitioners in Singapore and other Asian contexts. If the previous finding that violent offenders are a more severe group compared to nonviolent offenders is replicated (e.g., Baglivio, Jackowski, & Greenwald, 2014; Mulder et al., 2012), it warrants a sharper demarcation in intensity of treatment and amount of resources allocated between these two groups.

### Subtypes of Violent Youth Offenders

Loeber and Stouthamer-Loeber (1998) postulated that youth who are on the overt developmental pathway will show progression specialized in aggression and violence. In addition, some youth can be on multiple pathways, such as both the overt and the covert pathways (covert pathway involves concealing behaviors such as burglary and theft) or move between pathways (Loeber & Hay, 1994). This suggests a subtype specializing in violent offending and a subtype that is versatile, engaging in both violent and nonviolent offending.

With regard to the specialization/versatility of violent delinquents, the extant criminal career literature presents a mixed picture. The question of whether there is specialization in particular types of offending behavior is important because it determines whether specificity is needed in rehabilitation (e.g., targeted treatment and selective detention). Although many researchers had strongly purported that youth offending is predominantly versatile (e.g., Brame, Mulvey, Piquero, & Schubert, 2014; Farrington, Synder, & Finnegan, 1988; Piquero, 2000), there has also been evidence of specialization in violence (e.g., Besemer, 2012; Osgood & Schrek, 2007; Thomas, 2013). Resolution of the contrasting literature has been difficult, due to the lack of agreement about the definition of specialization and shortcomings in methods of analyzing for the presence of specialization (e.g., Baker, Metacalfe, & Jennings, 2013; Osgood & Schrek, 2007; Thomas, 2013). An even more complex picture has emerged, with research showing that specialization/versatility can vary across individual characteristics such as peer influence and marriage (McGloin, Sullivan, Piquero, & Pratt, 2007; Thomas, 2015) or situational variables such as alcohol influence (McGloin et al., 2007). Therefore, criminal career research and the closely related field of offender typology have to examine specialization/versatility further.

The question of specialization can also be addressed by testing for profile differences between youth offenders categorized as specialized violent offenders and those categorized as versatile violent offenders. The reasoning is that if different profiles are observed, then there is evidence for a specialized group and a versatile group. This simple approach may provide a less ambiguous set of findings on the existence of specialization, to augment criminal career research. Subtypes of violent offenders have not received much attention in typology studies and have been examined in only a handful of studies (Colins, Vermeiren, Schuyten, & Broekaert, 2009; Mulder et al., 2012; Smart et al., 2003; Vaughn, Salas-Wright, DeLisi, & Maynard, 2014). Generally, versatile violent youth offenders have a greater number of risk factors compared to specialized violent youth offenders (Smart et al., 2003; Vaughn et al., 2014). Even fewer studies have gone beyond risk factors to examine recidivism of the groups. For example, Mulder and colleagues found that versatile violent youth offenders have higher recidivism compared to the violence specialists. None of the studies used actuarial risk assessment tools to characterize the groups.

### Risk and Criminogenic Needs

One practical framework which can be used to examine violent offender typology is the risk, need, and responsivity (RNR) framework (Andrews, Bonta, & Hoge, 1990) considering its utility in offender rehabilitation (see meta-analyses by Andrews et al., 1990; Hanson, Bourgon, Helmus, & Hodgson, 2009). The RNR framework and its actuarial risk assessment tool, Youth Level of Service/Case Management Inventory (YLS/CMI), have previously been used in studies examining heterogeneity in other offender types, such as youth sex offenders (Zeng, Chu, Koh, & Teoh, 2015) and gang and nongang affiliated youth offenders (Chu, Daffern, Thomas, & Lim, 2012).

The RNR framework rests on three core tenets: (i) the intensity of the intervention should match the level of risk, (ii) criminogenic needs that are malleable should be targeted to reduce the risk of reoffending, and (iii) intervention delivery should be tailored according to an offender’s learning style and abilities for higher responsivity (Andrews et al., 1990). Identification of risk level and relevant criminogenic needs associated with each offender subtype informs the type and level of intervention needed (Clements, 1996). It is also useful to examine noncriminogenic needs, which have implications for the tailoring of interventions to ensure responsivity.

### Juvenile Justice in Singapore

Singapore is an independent island state in South East Asia with a population of 5.5 million (Singapore Department of Statistics, 2015). Many statutes are based on the English common law (e.g., the Criminal Procedure Code, 2012), but there are some statutes that are based on legislation from other jurisdictions; for example, the Penal Code (2008) is based on the Indian Penal Code, which was (nonetheless) first formulated by the English. As such, there are similarities in the way that offenses are defined when compared with the above-mentioned countries. Pertaining to crime statistics, youth arrests account for about 10% of all arrests in Singapore (Singapore Police Force, 2015).

Not all young offenders who commit offenses are charged in the juvenile court (Ministry of Social & Family Development, 2014). In line with a graduated approach in juvenile justice, first-time offenders who have committed minor crimes may be placed on diversionary programs. For those who are charged and convicted by the court, they may be required to undergo community rehabilitation which often includes probation or required to reside in a Juvenile Rehabilitation Centre (custodial facility for youth offenders) for a period of time.

In the early 2000s, there was a collective move by the youth and adult correctional services toward using structured and empirically informed approach to assess youth offenders’ risk and needs. The RNR framework was introduced to provide a theoretical and empirical-based approach to conduct offender assessment and rehabilitation. The YLS/CMI, and subsequently the YLS/CMI 2.0, was chosen as the primary risk assessment measure to assess the risk and needs of youth offenders (J. R. Chua, Chu, Yim, Chong, & Teoh, 2014). Within the Singaporean context, YLS/CMI 2.0’s coding criteria and cutoffs for risk categories were modified and developed, respectively, for assessing the local youth offenders and in accordance with the local legislation and procedures.

### Present Study

This study compared nonviolent youth offenders with violent youth offenders who had committed violent offenses only (*violent only*) and who had committed both violent and nonviolent offenses (*violent plus*) on YLS/CMI total risk scores, criminogenic and noncriminogenic needs, and risk of recidivism. In addition to overall recidivism, common forms of recidivism, violent, nonviolent, and sexual were examined. The aim was to determine whether violent youth were a different type from nonviolent youth based on differences in risk levels, rehabilitation needs, and reoffending patterns. By further differentiating violent youth, this study also explored whether there were subtypes of violent youth offenders (i.e., a specialized group and a versatile group).

Based on previous findings of a higher risk profile of violent offenders in contrast with nonviolent offenders (e.g., Baglivio et al, 2014; Mulder et al., 2012), it was hypothesized that violent youth offenders (i.e., violent only and violent plus) would have higher YLS/CMI risk scores and criminogenic needs, higher frequency of noncriminogenic needs, and higher risk of reoffending as compared to nonviolent youth offenders. However, the pattern of differences in risk and needs between violent only and nonviolent youth and between violent plus and nonviolent youth were expected to differ (Mulder et al., 2012; Vaughn et al., 2014). This study also sought to examine the hypothesis that violent-only youth offenders would differ from violent-plus youth offenders on risk and needs.

## Method

### Participants

The sample comprised of 3,744 youth who were charged with criminal offenses between January 2004 and December 2008 and were referred to the Ministry of Social and Family Development for either probation within the community or residence at a Juvenile Rehabilitation Centre (custodial facility for youth offenders). This sample represented 96.9% (3,264 of 3,370) of the youth on probation and 99.0% (480 of 485) of youth under custodial orders for that period of time; the remaining cases could not be coded as a result of missing information or file retrieval difficulties. There were 3,327 males (88.9%) and 417 females (11.1%) who were between 12 and 18 years old at the point of referral (*M* = 15.31, *SD* = 1.21).

As this study was specifically interested in violent nonsexual offenders (who are typologically different from sexual offenders; e.g., Craig, Browne, Beech, & Stringer, 2006; Van Wijk et al., 2006), 2.3% (87 of 3,744) of the sample who had committed sexual offenses were excluded.

In terms of index offenses committed, 67.1% (2,454 of 3,657) had committed nonviolent offenses only, 19.9% (727 of 3,657) had committed violent offenses only, and 13.0% (476 of 3,657) had committed both nonviolent and violent offenses. A small proportion, 2.4% (89 of 3,657), had records of prior offenses, while 10.9% (399 of 3,657) had subsequently breached their probation or custodial orders.

### Procedure

Approval for the study was obtained from the Ministry of Social and Family Development before commencing with data collection. Two psychologists, one probation officer, and five research assistants reviewed and coded participants’ case files retrospectively, between January 2011 and September 2012. The case files were obtained from the Ministry of Social and Family Development and consisted of (a) psychological reports, (b) presentence reports, (c) charge sheets, (d) statement of facts, (e) any previous assessment and treatment reports, and (d) school reports. Psychological and presentence reports contained detailed information pertaining to several key areas: personal, family, psychiatric, and criminal offending histories as well as the current offending behaviors and risk management issues. Prior to the study, the raters had completed a 3-day training by accredited trainers on administering YLS/CMI. They were also briefed on the procedures in reviewing and coding the case files.

Disagreements in coding were resolved through discussions. Interrater reliability for YLS/CMI ratings was assessed by the raters separately coding a random sample of 31 files. The intraclass correlation coefficient (ICC) for single rater (using absolute agreement definition; ICC) was .63 (*good*) for the YLS/CMI total score (see Cicchetti & Sparrow, 1981, for classification of ICCs). The ICCs for the eight domains were .43 (*fair*) for prior or current offenses/dispositions, .50 (*fair*) for family circumstances/parenting, .60 (*good*) for education/employment, .50 (*fair*) for peer relations, .49 (*fair*) for substance abuse, .45 (*fair*) for leisure/recreation, .55 (*fair*) for personality/behavior, and .48 (*fair*) for attitudes/orientation.

### Measures

#### Offender group

Our study adopted Farrington’s (1998) definition for violent acts, which is behavior intended to cause, or intended to and actually causes injury. Youth were categorized as *nonviolent offenders* if their index offenses comprised only offenses without the intent to cause injury, such as theft, fraud, and drug use. Those who had committed violent offenses exclusively, such as physical assault, rioting, and robbery, were categorized as *violent-only offenders*. Violence against animals or property was not included (Farrington, 1998). None of the youth were convicted of family violence. Those who had committed both violent and nonviolent offenses were categorized as *violent-plus offenders*.

#### Risk and criminogenic needs

The localized version of YLS/CMI 2.0 was used to code for *YLS/CMI total risk scores*, which is the risk of reoffending and the eight criminogenic needs (*prior or current offenses/dispositions, family circumstances/parenting, education/employment, peer relations, substance abuse, leisure/recreation, personality/behavior, and attitudes/orientation*). A total of 42 items on the domains prior or current offenses/dispositions (e.g., “three or more prior convictions”), family circumstances/parenting (e.g., “inadequate supervision”), education/employment (e.g., “low achievement”), peer relations (e.g., “some delinquent friends”), substance abuse (e.g., “chronic drug use”), leisure/recreation (e.g., “could make better use of time”), personality/behavior (e.g., “physically aggressive”), and attitudes/orientation (e.g., “antisocial/procriminal attitudes”) were rated as either *yes* or *no*. The total number of endorsed items on each domain reflects the level of domain-specific criminogenic needs. The YLS/CMI total risk scores were computed by summing up the domain scores. Previous meta-analyses showed that the YLS/CMI assessment ratings had modest to moderate predictive validity (see Olver, Stockdale, & Wormith, 2009; Schwalbe, 2007, for reviews). It also had moderate predictive validity for use in the Singaporean context (Chu et al., 2015). There have been fewer studies on the reliability of YLS/CMI, but there is evidence of fair to excellent interrater reliability of the domain scores and total score (Rocque & Plummer-Beale, 2014; Schmidt, Hoge, & Gomes, 2005).

#### Noncriminogenic needs

The YLS/CMI assessment also has a 51-item section that assesses the presence of noncriminogenic needs for individualized intervention (Andrews et al., 1990); items are rated as “yes” or “no.” Only the following items were coded as the required information was consistently collected and recorded in the reports: *chronic history of offenses, financial/accommodation problems, history of bullying, history of running away, history of sexual/physical assault, peers outside age range, poor problem solving skills, poor social skills, underachievement*, and *gang membership*.

#### Age at first charge

Age of the youth at first charge was derived using birth date and date of the first court charge.

#### Recidivistic outcomes

Recidivism data were obtained only after the completion of coding of all the other variables. The cutoff date for recidivism was April 20, 2011. The youth was considered to have *general recidivism*, if there had been either a conviction of any offense, violent, sexual, nonviolent, or other types of offenses, following the initial court order, or a breach of court orders. *Violent recidivism* (e.g., physical assault, rioting, and robbery), *nonviolent recidivism* (e.g., theft, fraud, and drug use), and *sexual recidivism* (e.g., indecent exposure, molestation, and peeping) were also measured.

### Statistical Analyses

All analyses were conducted using SPSS version 19.0. Comparisons were made between violent-only and violent-plus groups and the reference group, nonviolent. Comparisons were also made between violent-only and violent-plus groups. Continuous dependent variables were analyzed using MANOVA, followed by univariate tests with adjustment of Type I error using Benjamini and Hochberg’s procedure (1995). This procedure of adjustment for false discovery rate (FDR) is more powerful than adjustment for familywise error rates. Following significant univariate tests, post hoc comparisons were conducted using Hochberg’s GT2 to control for Type I error (Field, 2013). Categorical dependent variables were analyzed using χ^2^, likewise with the control of FDR.

To analyze whether group membership predicted differences in general recidivism, and specific types of recidivism, Cox regressions were conducted, which can account for differences in follow-up period for recidivism among the youth.

## Results

### Descriptives

Table 1 details the breakdown of the nonviolent, violent-only, and violent-plus groups by sex, ethnicity, current offense(s), and recidivism. The nonviolent group committed 2.84 offenses on average, while the violent-only group committed 1.54 violent offenses on average; the violent-plus group committed 2.74 nonviolent offenses on average and 1.62 violent offenses on average. The mean follow-up period for recidivism was 1,425 days (median = 1,382, *SD* = 668, range = 8–2,666).

**Table 1.** Descriptive Data for the Nonviolent, Violent-Only, and Violent-Plus Groups.

	Nonviolent (*n* = 2,454)	Violent Only (*n* = 727)	Violent Plus (*n* = 476)
Sex			
Males	2,162/2,454 (88.1%)	659/727 (90.6%)	421/476 (88.4%)
Females	292/2,454 (11.9%)	68/727 (9.4%)	55/476 (11.6%)
Ethnicity			
Chinese	1,250/2,454 (50.9%)	385/727 (53.0%)	261/476 (54.8%)
Indian	222/2,454 (9.0%)	60/727 (8.3%)	63/476 (13.2%)
Malay	859/2,454 (35.0%)	241/727 (33.1%)	133/476 (27.9%)
Eurasian	13/2,454 (0.5%)	2/727 (0.3%)	3/476 (0.6%)
Others	110/2,454 (4.5%)	39/727 (5.4%)	16/476 (3.4%)
Current general offending			
Mean	2.84	1.54	4.37
Median	2.00	1.00	3.00
*SD*	3.21	1.16	5.22
Range	1–40	1–15	2–81
Current violent offending			
Mean	Not applicable	1.54	1.62
Median	Not applicable	1.00	1.00
*SD*	Not applicable	1.16	1.43
Range	Not applicable	1–15	1–22
Current nonviolent offending			
Mean	2.84	Not applicable	2.74
Median	2.00	Not applicable	2.00
*SD*	3.21	Not applicable	4.83
Range	0–40	Not applicable	1–78
Recidivism			
General recidivism	947/2,454 (38.6%)	253/727 (34.8%)	239/476 (50.2%)
Violent recidivism	258/2,454 (10.5%)	88/727 (12.1%)	80/476 (16.8%)
Nonviolent recidivism	859/2,454 (35.0%)	214/727 (29.4%)	210/476 (44.1%)
Sexual recidivism	9/2,454 (0.4%)	3/727 (0.4%)	2/476 (0.4%)

### Group Differences in Risk and Criminogenic Needs

Table 2 displays the means and standard deviations of the nonviolent, violent-only, and violent-plus groups on the YLS/CMI. An MANOVA was conducted with group as the independent variable, and scores of the YLS/CMI (total and domain), and age at first charged offense as dependent variables, to explore for differences between the three groups. There was a main effect of group type on the MANOVA, *F*(20, 7,278) = 23.70, *p* < .001, *partial* η^2^ = .06, indicating differences among all three groups. After applying an FDR adjustment, tests of between-subjects effects revealed that differences were present among all dependent variables (see Table 2).

**Table 2.** Means and Standard Deviation of YLS/CMI for the Nonviolent, Violent-only, and Violent-Plus Groups.

YLS/CMI		Nonviolent (*n* = 2,449)	Violent Only (*n* = 726)	Violent Plus (*n* = 475)	*df*	*F*	*p*	Partial η^2^
YLS/CMI total score	*M*	12.29	12.42	14.31	(2, 3,647)	41.63	<.001^b,c^	.022
	*SD*	4.53	4.13	4.50				
Prior and current offenses/dispositions	*M*	0.23	0.07	0.42	(2, 3,647)	88.57	<.001^a,b,c^	.046
	*SD*	0.46	0.33	0.56				
Family circumstances/parenting	*M*	2.29	2.13	2.48	(2, 3,647)	11.82	<.001^a,b,c^	.006
	*SD*	1.26	1.19	1.20				
Education/employment	*M*	2.12	2.03	2.52	(2, 3,647)	15.66	<.001^b,c^	.009
	*SD*	1.58	1.61	1.70				
Peer relations	*M*	3.06	3.36	3.39	(2, 3,647)	34.99	<.001^a,b^	.019
	*SD*	1.13	0.92	0.95				
Substance abuse	*M*	0.24	0.17	0.35	(2, 3,647)	11.77	<.001^a,b,c^	.006
	*SD*	0.64	0.48	0.80				
Leisure/recreation	*M*	2.26	2.29	2.42	(2, 3,647)	5.61	.004^b^	.003
	*SD*	0.95	0.92	0.82				
Personality/behavior	*M*	0.81	1.24	1.27	(2, 3,647)	70.39	<.001^a,b^	.037
	*SD*	1.05	1.06	1.09				
Attitudes/orientation	*M*	1.29	1.14	1.47	(2, 3,647)	17.35	<.001^a,b,c^	.009
	*SD*	0.93	0.85	1.02				
Age at first charge	*M*	15.20	15.27	14.97	(2, 3,647)	8.78	<.001^b,c^	.005
	*SD*	1.26	1.24	1.31				

^a^Significant difference between nonviolent and violent only. ^b^Significant difference between nonviolent and violent plus. ^c^Significant difference between violent only and violent plus.

Hochberg’s GT2 post hoc tests revealed that scores were significantly higher for the nonviolent group when compared to the violent-only group on the YLS/CMI domains of prior and current offenses/dispositions (*p* < .001), family circumstances/parenting (*p* = .006), substance abuse (*p* = .036), and attitudes/orientation (*p* < .001); scores were significantly higher for the violent-only group as compared to the nonviolent group on domains of peer relations (*p* < .001) and personality/behavior (*p* < .001).

Post hoc tests also revealed that youth in the violent-plus group were rated significantly higher on the total score and all domains of the YLS/CMI as compared to those in the nonviolent group (*p*s from <.001 to .002). Additionally, youth in the violent-plus group were younger at the age of first charge than those in the nonviolent group (*p* < .001).

Finally, youth in the violent-plus group were rated significantly higher than violent-only youth for the domains of prior and current offenses/dispositions, family circumstances/parenting, education/employment, substance abuse, and attitudes/orientation, as well as the total score (*p*s < .001). Additionally, youth in the violent-plus group were younger at the age of first charge than those in the violent-only group (*p* < .001).

### Group Differences in Noncriminogenic Needs

Group comparisons for noncriminogenic needs were also carried out using Pearson’s χ^2^ tests. Three tests were conducted to compare (a) the nonviolent and violent-only groups, (b) the nonviolent and violent-plus groups, and (c) the violent-only and violent-plus groups.

From Table 3, after FDR adjustments, results indicated that violent-only youth were more likely than nonviolent youth to have histories of sexual/physical assault (OR = 2.22, *p* < .001), poor problem-solving skills (OR = 1.50, *p* < .001), and be a gang member (OR = 1.87, *p* < .001).

**Table 3.** Associations between Nonviolent and Violent-Only Groups on Noncriminogenic Needs.

Noncriminogenic Needs	Nonviolent	Violent Only	*p*	Φ	*OR* (Violent Only/Nonviolent)
	*n* (%)	*n* (%)			
Chronic history of offenses	528/2,454 (21.5%)	139/727 (19.1%)	.163	−.025	
Financial/accommodation problems	509/2,454 (20.7%)	124/727 (17.1%)	.029	−.039	
History of bullying	229/2,454 (9.3%)	63/727 (8.7%)	.585	−.010	
History of running away	329/2,454 (13.4%)	79/727 (10.9%)	.072	−.032	
History of sexual/physical assault	450/2,454 (18.3%)	242/727 (33.3%)	<.001^†^	.152	2.22
Peers outside age range	335/2,454 (13.7%)	90/727 (12.4%)	.376	−.016	
Poor problem-solving skills	684/2,454 (27.9%)	267/727 (36.7%)	<.001^†^	.081	1.50
Poor social skills	155/2,454 (6.3%)	52/727 (7.2%)	.442	.014	
Underachievement	630/2,454 (25.7%)	186/727 (25.6%)	.962	−.001	
Gang membership	581/2,454 (23.7%)	267/727 (36.7%)	<.001^†^	.124	1.87

*Note*. *OR* is only displayed for significant χ^2^ tests. *OR =* odds ratio.

^†^Significant after false discovery rate correction. *Significant at .05 level.

From Table 4, violent-plus youth were more likely than nonviolent youth to have had histories of bullying (OR = 1.65, *p* < .001) and sexual/physical assault (OR = 2.28, *p* < .001). Violent-plus youth were also more likely to have poor problem-solving skills (OR = 1.76, *p* < .001) and be a gang member (OR = 2.55, *p* < .001).

**Table 4.** Associations Between Nonviolent and Violent-Plus Groups on Noncriminogenic Needs.

Noncriminogenic Needs	Nonviolent	Violent Plus	*p*	Φ	*OR* (Violent Plus/Nonviolent)
	*n* (%)	*n* (%)			
Chronic history of offenses	528/2,454 (21.5%)	111/476 (23.3%)	.383	.016	
Financial/accommodation problems	509/2,454 (20.7%)	109/476 (22.9%)	.291	.020	
History of bullying	229/2,454 (9.3%)	69/476 (14.5%)	<.001^†^	.063	1.65
History of running away	329/2,454 (13.4%)	80/476 (16.8%)	.050	.036	
History of sexual/physical assault	450/2,454 (18.3%)	162/476 (34.0%)	<.001^†^	.142	2.28
Peers outside age range	335/2,454 (13.7%)	64/476 (13.4%)	.905	−.002	
Poor problem-solving skills	684/2,454 (27.9%)	193/476 (40.5%)	<.001^†^	.102	1.76
Poor social skills	155/2,454 (6.3%)	31/476 (6.5%)	.872	.003	
Underachievement	630/2,454 (25.7%)	135/476 (28.4%)	.222	.023	
Gang membership	581/2,454 (23.7%)	210/476 (44.1%)	<.001^†^	.170	2.55

*Note*. *OR* is only displayed for significant χ^2^ tests. *OR =* odds ratio.

^†^Significant after false discovery rate correction. *Significant at .05 level.

Finally, violent-plus youth were more likely than violent-only youth to have histories of bullying (OR = 1.79, *p* = .002) and running away (OR = 1.66, *p* = .002; see Table 5). They were also more likely to have familial financial/accommodation difficulties (OR = 1.44, *p* = .012) and gang membership (OR = 1.36, *p* = .010).

**Table 5.** Associations Between Violent-Only and Violent-Plus Group on Noncriminogenic Needs.

Noncriminogenic Needs	Violent Only	Violent Plus	*p*	Φ	*OR* (Violent Plus/Violent Only)
	*n* (%)	*n* (%)			
Chronic history of offenses	139/727 (19.1%)	111/476 (23.3%)	.079	.051	
Financial/accommodation problems	124/727 (17.1%)	109/476 (22.9%)	.012^†^	.072	1.44
History of bullying	63/727 (8.7%)	69/476 (14.5%)	.002^†^	.091	1.79
History of running away	79/727 (10.9%)	80/476 (16.8%)	.003^†^	.086	1.66
History of sexual/physical assault	242/727 (33.3%)	162/476 (34.0%)	.789	.008	
Peers outside age range	90/727 (12.4%)	64/476 (13.4%)	.589	.016	
Poor problem-solving skills	267/727 (36.7%)	193/476 (40.5%)	.182	.038	
Poor social skills	52/727 (7.2%)	31/476 (6.5%)	.668	−.012	
Underachievement	186/727 (25.6%)	135/476 (28.4%)	.287	.031	
Gang membership	267/727 (36.7%)	210/476 (44.1%)	.010^†^	.074	1.36

*Note*. *OR* is only displayed for significant χ^2^ tests. *OR =* odds ratio.

^†^Significant after false discovery rate correction. *Significant at .05 level.

### Group Differences in Recidivistic Outcomes

Cox regressions were conducted to ascertain whether group membership predicted general, violent, nonviolent, and sexual recidivism while taking into account the time at risk.^1^ Results indicated that the models predicting various types of recidivism from group membership were significant, with the exception of sexual recidivism (see Table 6). From Table 6, violent-plus youth were more likely than nonviolent youth to reoffend generally (HR = 1.40, 95% CI [1.22, 1.62], *p* < .001), violently (HR = 1.64, 95% CI [1.28, 2.11], *p* < .001), and nonviolently (HR = 1.36 times, 95% CI [1.17, 1.58], *p* < .001). They were also more likely than violent-only youth to reoffend generally (HR = 1.59, 95% CI [1.33, 1.90], *p* < .001), violently (HR = 1.41, 95% CI [1.04, 1.90], *p* = .028), and nonviolently (HR = 1.69, 95% CI [1.39, 2.04], *p* < .001). Violent-only youth were less likely than nonviolent youth to commit nonviolent (HR = 0.81, 95% CI [0.69, 0.94], *p* = .005) reoffending. There were no significant differences between violent-only and nonviolent youth in their likelihood of general and violent reoffending.

**Table 6.** Prediction of Recidivism Type from Groups (Cox Regression).

Recidivism Type	*B*	*SE*	Wald	*df*	*p*	HR	95% CI
General recidivism			29.43	2	<.001		
Violent-only vs. nonviolent	−.13	.07	3.10	1	.078	0.88	[0.77, 1.01]
Violent-plus vs. nonviolent	.34	.07	21.69	1	<.001	1.42	[1.22, 1.62]
Violent-plus vs. violent-only	.46	.09	26.23	1	<.001	1.59	[1.33, 1.90]
Violent recidivism			15.11	2	.001		
Violent-only vs. nonviolent	.16	.12	1.59	1	.207	1.17	[0.92, 1.49]
Violent-plus vs. nonviolent	.50	.13	15.00	1	.000	1.64	[1.28, 2.11]
Violent-plus vs. violent-only	.34	.15	4.84	1	.028	1.41	[1.04, 1.90]
Nonviolent recidivism			29.38	2	<.001		
Violent-only vs. nonviolent	−.22	.08	8.03	1	.005	0.81	[0.69, 0.94]
Violent-plus vs. nonviolent	.31	.08	15.75	1	<.001	1.36	[1.17, 1.58]
Violent-plus vs. violent-only	.52	.10	28.88	1	<.001	1.69	[1.39, 2.04]
Sexual recidivism			0.45	2	.978		
Violent-only vs. nonviolent	.11	.67	0.29	1	.864	1.12	[0.30, 4.14]
Violent-plus vs. nonviolent	.13	.78	0.25	1	.873	1.13	[0.25, 5.24]
Violent-plus vs. violent-only	.01	.91	0	1	.991	1.01	[0.17, 6.05]

## Discussion

The present study examined differences in the profile of risk and rehabilitation needs of youth who offended nonviolently, only violently, and both nonviolently and violently, to establish if there were different types of youth offenders. Overall, our data largely supported the hypotheses and confirmed that violent and nonviolent youth offenders were a valid typological distinction. Violent-plus youth had higher total risk scores and criminogenic needs on all domains, were more likely to have several noncriminogenic needs, and were consistently at higher risk of reoffending across all types of reoffending except sexual reoffending, when compared to nonviolent youth. Despite having the same total risk scores and risk of general and violent recidivism as nonviolent youth, violent-only youth presented a different set of criminogenic and noncriminogenic needs, and risk of nonviolent recidivism. There was also support for the exploratory hypothesis that violent-only youth offenders and violent-plus youth offenders were distinct types.

### Typological Distinction Between Violent and Nonviolent Youth Offenders

Clearly, the violent-plus youth were a higher risk group when compared to the nonviolent youth. Violent-plus youth were more likely to have history of aggression (i.e., bullying and sexual/physical assault) and poor problem-solving skills. Violent-plus youth were also more likely to be involved in gangs than their nonviolent counterparts. These findings are in line with literature showing differences in the risk factors for violent youth vis-à-vis nonviolent youth and associations between risk factors and violence (Baglivio et al., 2014; Mulder et al., 2012; Valois, MacDonald, Bretous, Fischer, & Drane, 2002; Zhou et al., 2014). The findings on total risk scores and recidivistic outcomes also parallel findings of higher risk scores and reoffending behavior in previous studies (Baglivio et al., 2014; Chu et al., 2012; Mulder et al., 2012).

Likewise, violent-only and nonviolent youth had differing risk and needs, and risk of nonviolent recidivism, though there were fewer number of differences compared to the comparisons of violent-plus and nonviolent youth. The consistent differences between the nonviolent youth and the two variants of violent youth on a number of profile characteristics suggest that it is valid to distinguish youth offenders on the basis of whether they offended violently. The findings offer additional evidence for violent offender types (Loeber & Farrington, 1998; Loeber & Stouthamer-Loeber, 1998) and the premise that offenders are heterogeneous (Clements, 1996; Moffitt, 1993) in a culturally distinctive, non-Western youth offender sample. Beyond profile differences, the findings also imply that the level of effective dosage and the appropriate targets of intervention vary between violent and nonviolent youth offenders. For example, in line with the risk principle of RNR (Andrews et al., 1990), the intensity of intervention to reduce reoffending among violent-plus youth should be higher relative to that needed for nonviolent youth. According to the need principle, intervention to address pertinent criminogenic needs should reduce negative peer influence for violent-only youth but not for nonviolent youth.

### Subtypes of Violent Youth Offenders

There is preliminary evidence for subtypes of violent youth offenders. The pattern of differences in the comparisons between violent-only and nonviolent youth and between violent-plus and nonviolent youth was largely dissimilar. The only parallels were higher needs on the peer relations and personality/behavior domains and higher likelihood of being involved in gangs, having poor problem-solving skills, and history of sexual/physical assault.

More importantly, differences were found in the direct comparisons between the violent-plus and the violent-only groups. Violent-plus youth had higher total risk scores and criminogenic needs on five domains and were at higher risk of general, violent, and nonviolent reoffending. No differences were observed for sexual reoffending (like what was found for comparisons between these two groups and nonviolent youth), which is not surprising, given the overall low base rate of sexual reoffending in this sample. Furthermore, low rates of sexual reoffending have been found among Singaporean youth offenders, even for youth who have sexually offended (Zeng, Chu, & Lee, 2015). Violent-plus youth were also more likely to have history of delinquent behavior (i.e., running away), history of aggression (i.e., bullying), and be involved in gangs. These findings are significant because (i) it confirms the typology literature on violent subtypes (Loeber & Stouthamer-Loeber, 1998, Mulder et al., 2012; Smart et al., 2003; Vaughn et al., 2014) and (ii) it provides evidence for specialization in violent offending behavior (Besemer, 2012; Osgood & Schrek, 2007; Thomas, 2013). It is also interesting that profile differences in line with what past studies have found between groups constructed from delinquent records accumulated over a duration of delinquent careers (Smart et al., 2003; Vaughn et al., 2014) were observed between groups comprising mostly of first-time offenders. This raises the possibility that specialized violent offenders and versatile violent offenders may be identified as early as at the point of their first convictions.

Overall, violent-plus youth, or the criminally versatile, appear to be the most high-risk offender type among the three groups. In addition, the observation of lowest age at first charge among versatile violent youth suggests that they would be at risk of persistent future offending (Gann, Sullivan, & Ilchi, 2015), though it is noted that the age differences between them and the other two groups were very small. Findings imply that rehabilitation for versatile violent youth needs to be administered at a high intensity and also incorporate program that break the cycle of continuous offending.

### Strengths and Limitations

The present study has a number of strengths and limitations. Strengths include a large, representative youth offender sample across several cohorts, a non-Western sample, the inclusion of breach during court orders in the measure of recidivism to minimize underestimation of reoffending among those who were on custodial orders, and the use of time to recidivism, a more sensitive measure of recidivism than other measures (Maltz, 1984).

The use of a large number of coders to conduct coding may have affected the effect sizes and ICCs. Using Cohen’s guidelines (1988), the effect sizes ranged from small to moderate. The size of offender group differences on risk and criminogenic needs was either small or moderate, with *r* ranging from .06 to .36. The strength of associations between offender group and risk factors was small, with Φ ranging from .05 to .17. Although it was expedient to have many coders for such a large sample, it could have introduced more variance to the data. Likewise, the ICCs of YLS/CMI could have been affected and were mostly fair according to Cicchetti and Sparrow’s guidelines (1981).

Since this was a retrospective study conducted after youth offenders have exited the justice system, there was no information on self-reported offending behavior. Therefore, time to recidivism could not account for instances of reoffending which had gone undetected by authorities. In addition, without data on self-reported offending prior to the current offense, the frequency of offending could not be reliably measured among these first-time entrants to the justice system and controlled for (e.g., Piquero, 2000). Farrington, Snyder, and Finnegan (1988) had cautioned that “despite problems of concealment and forgetting, self-reports may be more useful than official records in studies of specialization based on numbers of different types of offenses committed during offending careers” (p. 467).

Another limitation of the study is that the majority of the sample comprised male youth offenders. Caution should be exercised in generalizing the findings to female youth offenders, given that there are gender differences in aggression, the development of aggression (Loeber & Stouthamer-Loeber, 1998), and risk factors for aggression (Penney, Lee, & Moretti, 2010; Stephenson, Woodhams, & Cooke, 2014; Valois et al., 2002). It would be useful for future studies to examine violent offender typology among female youth offenders.

## Note

1. A note should be made about the risk of reoffending of youth offenders residing in Juvenile Rehabilitation Centres. Their risk of reoffending during their custodial orders was comparable with youth offenders on probation, since breaches of orders were included as recidivistic outcomes; 11.9% (55 of 461) had breached their initial orders, whereas 51.6% (238 of 461) had reoffended during the follow-up period. For youth offenders on probation, 10.8% (344 of 3,196) had breached their initial orders, whereas 37.6% (1,201 of 3,196) had reoffended during the follow-up period.
